# Blood analytes of immature Kemp’s ridley sea turtles (*Lepidochelys kempii*) from Georgia, USA: reference intervals and body size correlations

**DOI:** 10.1093/conphys/coaa091

**Published:** 2020-12-01

**Authors:** Justin R Perrault, Michael D Arendt, Jeffrey A Schwenter, Julia L Byrd, Craig A Harms, Carolyn Cray, Kathryn A Tuxbury, Lawrence D Wood, Nicole I Stacy

**Affiliations:** 1 Loggerhead Marinelife Center, Juno Beach, Florida, 33408, USA; 2 Marine Resources Division, South Carolina Department of Natural Resources, Charleston, South Carolina, 29412, USA; 3 South Atlantic Fish Management Council, North Charleston, South Carolina, 29405, USA; 4 Department of Clinical Sciences and Center for Marine Sciences and Technology, College of Veterinary Medicine, North Carolina State University, Morehead City, North Carolina, 27606, USA; 5 Division of Comparative Pathology, Department of Pathology and Laboratory Medicine, Miller School of Medicine, University of Miami, Miami, Florida, 33136, USA; 6 Animal Health Department, New England Aquarium, Central Wharf, Boston, Massachusetts, 02110, USA; 7 Florida Hawksbill Project at the National Save the Sea Turtle Foundation, Fort Lauderdale, Florida, 33308, USA; 8 Aquatic, Amphibian, and Reptile Pathology Program, Department of Comparative, Diagnostic, and Population Medicine, College of Veterinary Medicine, University of Florida, Gainesville, Florida, 32608, USA

**Keywords:** Health, in-water study, marine turtle, packed cell volume, plasma biochemistry, protein electrophoresis

## Abstract

Health assessments of wildlife species are becoming increasingly important in an ever-changing environment. Kemp’s ridley sea turtles (*Lepidochelys kempii*; hereafter, Kemp’s ridleys) are critically endangered and incur several on-going threats to their population recovery; therefore, it is imperative to advance the understanding of baseline blood analyte data as a diagnostic and monitoring tool. For in-water, trawl-captured, immature Kemp’s ridleys (minimum *N* = 31) from Georgia, USA, the objectives of this study were to (1) establish reference intervals (RIs) for packed cell volume (PCV) and 27 plasma biochemistry analytes and (2) determine length-specific relationships in blood analytes. We observed significant positive correlations between minimum straight carapace length and PCV, amylase, calcium:phosphorus ratio, cholesterol, magnesium, triglycerides, total solids, total protein and all protein fractions (e.g. alpha-, beta- and gamma-globulins); aspartate aminotransferase and chloride showed significant negative relationships. These results suggest that certain blood analytes in Kemp’s ridleys change as these animals grow, presumptively due to somatic growth and dietary shifts. The information presented herein, in due consideration of capture technique that may have impacted glucose and potassium concentrations, represents the first report of blood analyte RIs for Kemp’s ridley sea turtles established by guidelines of the American Society for Veterinary Clinical Pathology and will have direct applications for stranded individuals in rehabilitative care and for future investigations into the health status of wild individuals from this population.

## Teaser

Health assessments of endangered wildlife are essential for conservation and management, as populations cannot recover if the individuals within them are unhealthy. We analyzed blood analytes in immature Kemp’s ridley sea turtles, established reference intervals and identified changes in blood analytes associated with growth.

## Introduction

Blood analytes (e.g. hematology, biochemistry) are commonly investigated in wildlife species in an effort to further understand physiology and disease (Christopher *et al.*, 1999; Bounous *et al.*, 2000; Borjesson *et al.*, 2000; Geffré *et al.*, 2009; Flint *et al.*, 2010b; Kaufman *et al.*, 2018). These measures provide useful information regarding the physiological state and health status of an individual, such as nutritional and hydration status, electrolyte balance, reproductive state and organ system functions (Geffré *et al.*, 2009). Fifty years ago, the concept of reference intervals (RIs) was introduced in human medicine in an effort to understand changes in blood analytes, by defining health and disease through comparisons to ‘normal’ data of known apparently healthy individuals with refined inclusion criteria, a concept that has been adopted by veterinary medicine (Gräsbeck and Saris, 1969; Gräsbeck, 1983, 1990; Walton, 2001; Geffré *et al.*, 2009; Inoue *et al.*, 2012). These RIs are most commonly formulated by calculating the range that represents the central 95% of the reference population, with 90% confidence intervals of the lower and upper limits of the range also reported (Geffré *et al.*, 2009; Friedrichs *et al.*, 2012).

Logistical challenges (e.g. animal capture, sample size, confounding effects of stress and handling) occur with establishing RIs in threatened and endangered wildlife species in various settings (Wobeser, 2007; Ryser-Gegiorgis, 2013; Deem and Harris, 2017; Coogan *et al*., 2018). Therefore, RI establishment in wildlife populations is a developing field of study. RIs have been established in some sea turtle species and populations [leatherback sea turtles (*Dermochelys coriacea*); hereafter, leatherbacks; loggerhead sea turtles (*Caretta caretta*); hereafter, loggerheads; green turtles (*Chelonia mydas*)] (Aguirre and Balazs, 2000; Flint *et al.*, 2010a, b; Osborne *et al.*, 2010; Kelly *et al.*, 2015; Page-Karjian *et al.*, 2015, 2020; Stacy *et al.*, 2018a, 2019; Fleming *et al.*, 2020), yet no RIs exist for critically endangered Kemp’s ridley sea turtles [*Lepidochelys kempii*; hereafter, Kemp’s ridleys] (Wibbels and Bevan, 2019). Understanding baseline health of this sea turtle species is critical, as several on-going threats to their population recovery still exist (Wibbels and Bevan, 2019).

Nesting grounds for Kemp’s ridleys are mostly restricted to the western Gulf of Mexico (Texas, USA, to Veracruz, Mexico), with Rancho Nuevo, Tamaulipas, Mexico, experiencing the majority of nesting. Developmental and foraging habitats for Kemp’s ridleys produced on these beaches are located throughout the Gulf of Mexico and along the Atlantic coast of the United States (Wibbels and Bevan, 2019). Prior to the 2010 ‘Deepwater Horizon’ (DWH) oil spill, the Kemp’s ridley nesting population of Rancho Nuevo, Mexico, was experiencing an exponential increase. Since that disaster, there has been a deviation from this trend. Reasons for the decline remain unknown but could include fisheries interactions, poaching, pollution impacts from the DWH oil spill and/or carrying capacity of this species in the Gulf of Mexico (Gallaway *et al.*, 2016; McDonald *et al.*, 2017; Mitchelmore *et al.*, 2017; Wallace *et al.*, 2017; Caillouet *et al.*, 2018; Wibbels and Bevan, 2019). The DWH oil spill in the Gulf of Mexico provided significant justification for the need to establish RIs in this species, so that potential deviations in blood analytes may be recognized as a result of future environmental or physiological perturbations (e.g. natural disasters, disease, pollution impacts) (Stacy *et al.*, 2017). A number of studies have investigated blood analytes in Kemp’s ridleys (McNally *et al.*, 2020); however, these are usually in relation to some type of stressor including forced submergence (Stabenau *et al.*, 1991; Snoddy *et al.*, 2009), cold stunning (Carminati *et al.*, 1994; Turnbull *et al.*, 2000; Innis *et al.*, 2007, 2009), transport stress (Hunt *et al.*, 2016) or exposure to biotoxins and toxicants (Innis *et al.*, 2008; Perrault *et al.*, 2014, 2017). Additionally, blood analytes established in other Kemp’s ridley studies used captive, rehabilitating individuals exposed to dietary differences and other captivity effects (e.g. stress, water temperature differences, etc.) (Stabenau *et al.*, 1991; Moon *et al.*, 1999; Anderson *et al.*, 2011; Innis *et al.*, 2007, 2008, 2009; Coleman *et al.*, 2016; Hunt *et al.*, 2016, 2019). Therefore, extrapolation to wild populations should be done with appropriate caution (Bolten and Bjorndal, 1992). For in-water, trawl-captured, immature Kemp’s ridleys from Georgia, USA, the objectives of this study were to (1) establish RIs for blood analytes including packed cell volume (PCV) and 27 plasma biochemistry analytes and (2) determine length-specific relationships with blood analytes.

## Materials and Methods

### Ethical procedures

Our study was carried out in accordance with an Endangered Species Act Section 10(a)(1)(A) permit #19621, a Georgia Department of Natural Resources Scientific Collection Permit #CN21303 and an approved Institutional Animal Care and Use Committee protocol (UF IACUC# 201706823).

### Trawls

Sea turtle captures and sampling occurred from 31 May–15 Jul 2016 and 5 Jun–19 Jul 2017 and were conducted similar to methods (to include trawl gear) described in Arendt *et al.*, (2012a, b). Trawls occurred at 2.8 knots for ≤30 min between 4.6 m and 17.0 m depth. One Kemp’s ridley sea turtle was captured off Charleston, South Carolina, USA; all others were captured near Brunswick, Georgia, USA.

### Sea turtle capture, morphometrics, physical examination and sample collection and processing

Upon capture, Kemp’s ridley sea turtles were given a physical examination that consisted of visual evaluation of body condition, epibiota, external injuries and any other overt visible abnormalities. Mass (in kg) and minimum straight carapace length (SCL_min_, in cm) were recorded using a digital hanging scale (Pesola PSH200) and calipers, respectively. Body condition index (BCI) was calculated (Bjorndal *et al.*, 2000).

All captured Kemp’s ridleys were examined for internal and external tags; if neither of these tags were present, up to two Inconel flipper tags and an internal PIT tag (Biomark, Inc., Boise, Idaho USA) were applied prior to release (Florida Fish and Wildlife Conservation Commission, 2016). The tagging sites were disinfected with povidone iodine and isopropyl alcohol to prevent infection before and after tag application (Florida Fish and Wildlife Conservation Commission, 2016).

Following physical examinations, 10 ml of blood were collected from the dorsal cervical sinus (i.e*.* external jugular vein) of each turtle using a 21-gauge, 1.5″ needle and 10 ml sodium heparin vacutainer tubes (Becton Dickinson, Franklin Lakes, New Jersey, USA), following federal regulations regarding animal handling and sample collection (National Marine Fisheries Service Southeast Fisheries Science Center, 2008). The sampling site was swabbed with alternating applications of povidone iodine and alcohol prior to blood collection. A subsample of whole blood was aliquoted for PCV analysis (described below) and the remaining blood was centrifuged immediately (within <5 min) on the research vessel at 944 *g* (3600 rpm) for 5 min in a Clay Adams Sero-fuge™ centrifuge (Becton Dickinson, Sparks, Maryland, USA). Plasma was separated and stored in liquid nitrogen onboard until completion of multi-day overnight research cruises before storage in a shore-based ultralow freezer for up to 3 months prior to sample analysis, which is generally assumed to have no major effects on various analytes (Cray *et al.*, 2009; Townsend *et al*., 2020); however, potential artifactual changes cannot be completely ruled out (Thoresen et al. 1995).

### Analysis of blood analytes

Onboard the vessel, PCV was determined using whole blood drawn into microhematocrit tubes followed by centrifugation for 5 min at 13 000 g (11,500 rpm) using a micro-capillary centrifuge (Model MB, International Equipment Company, Needham Heights, Massachusetts USA). A hematocrit microcapillary tube reader was used to determine PCV as a percentage. Also onboard, total solids in plasma were estimated using a Westover Scientific RHC-200 ATC handheld refractometer (Woodinville, Washington, USA) and plasma color was visually assessed.

Frozen plasma samples were shipped overnight on dry ice to the University of Miami Avian and Wildlife Laboratory for biochemical analyses using an Ortho 250XR (Ortho Clinical Diagnostics, Rochester, New York, USA) dry slide chemistry analyzer. Biochemical analytes included alkaline phosphatase (ALP), amylase, aspartate aminotransferase (AST), blood urea nitrogen, calcium, chloride, cholesterol, creatine phosphokinase (CPK), gamma-glutamyl transferase (GGT), glucose, lipase, magnesium, phosphorus, potassium, sodium, total protein, triglycerides and uric acid. The calcium:phosphorus ratio was calculated.

Protein fractions were determined using the SPIFE 3000 system (Helena Laboratories Inc., Beaumont, Texas, USA). Fraction delimits were placed using the following conventions in electrophoretograms: pre-albumin, albumin, alpha_1_-globulins, alpha_2_-globulins, beta-globulins and gamma-globulins. Total globulins and the albumin:globulin ratio were calculated. For comparison to free-ranging, immature green turtles, loggerheads, leatherbacks and hawksbill sea turtles (*Eretmochelys imbricata*; hereafter, hawksbills) of the same life-stage class, representative electrophoretograms from each species using identical methodology for protein electrophoresis were chosen from other projects.

### Statistical analyses

Statistical analyses were performed using Medcalc® statistical software (version 19.1, Ostend, Belgium). Mean, standard error, standard deviation, median and range are reported for PCV and plasma biochemistry in standard international (SI) units. Length–mass relationships were assessed using power regression.

RIs (95% with associated 90% confidence intervals) were also calculated using parametric methods based on recommendations by Friedrichs *et al.* (2012) for sample sizes ≥20, but <40. Normality was assessed using the Shapiro–Wilk test (Shapiro and Wilk, 1965), while outliers were detected using the Dixon–Reed test (Reed *et al*., 1971). When appropriate, logarithmic transformations were employed and outliers were removed to generate accurate RIs. For CPK, RIs were calculated using the robust method, as data could not be normalized to fit a Gaussian distribution (i.e. parametric method).

Relationships between SCL_min_ and the measured blood analytes were determined using least-squares linear regressions. Because of the very strong correlation between SCL_min_ and mass (see Results section below), only SCL_min_ was used in analyses. Outliers were determined by Tukey’s test and were removed as appropriate. Data were transformed when necessary to meet the assumptions of the tests. The slopes of the lines of best fit between pre-albumin + albumin and globulins in relation to SCL_min_ were compared using a Student’s *t*-test (e.g. the slope of the line of best fit between pre-albumin + albumin and SCL_min_ was compared to the slope of the line of best fit between total globulins and SCL_min_).

## Results

### Physical examination and morphometrics

A total of 36 individual Kemp’s ridleys were captured from 31 May–15 July 2016 (*N* = 17) and 5 June–19 July 2017 (*N* = 19). A summary of mass, SCL_min_ and BCI are reported in Table 1. Three turtles were excluded from the length–mass analyses due to one individual having an abnormally shaped carapace (from an old healed shark predation lesion, 31 cm long × 8 cm wide, to the right posterior carapace), one individual missing a measurement for mass and another individual (the turtle caught in South Carolina) having monofilament fishing line wrapped around the neck and front flippers in addition to observed line extruding from the oral cavity, which likely impacted the ability to forage as shown by a comparatively low BCI (= 1.40). This animal was taken to the Sea Turtle Care Center™ at the South Carolina Aquarium for treatment.

**Table 1:** Mass and morphometric data from in-water Kemp’s ridley sea turtles (*Lepidochelys kempii*) from Georgia, USA (*N* = 33). Individuals with visible abnormalities (e.g. shark bites, monofilament line ingestion) were excluded from the following dataset

	Mass (kg)	SCL_min_ (cm)	BCI
Mean ± SE	13.6 ± 1.4	42.1 ± 1.6	1.62 ± 0.02
SD	8.2	9.1	0.09
Median	12.2	42.3	1.63
Range	2.6–35.0	26.2–61.5	1.45–1.90

**Figure 1:**
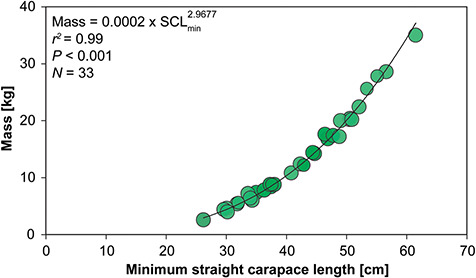
Relationship between mass (kg) and minimum straight carapace length (SCL_min_, cm) for in-water assessed Kemp’s ridley sea turtles (*Lepidochelys kempii*) from Georgia, USA. The equation for the line of best fit is shown.

Thirty-five of 36 turtles were considered to be of immature life-stage class based on SCL_min_ (range: 26.2–56.6 cm); one individual was determined to be an adult female (SCL_min_ = 61.5 cm) based on size estimates at maturity (breeding size of Kemp’s ridleys is ≥58 cm SCL_min_; Florida Fish and Wildlife Conservation Commission, 2016). Mass (in kg) and SCL_min_ (in cm) showed a very strong relationship (*r^2^* = 0.99; *P* < 0.001; Fig. 1).

Turtles (*N* = 35) included in this study were determined to be in good body condition based on visual observations of the thickness of neck and shoulder soft tissues and BCI scores of known healthy compared to emaciated sea turtles (Stacy *et al.*, 2018b). Minimal barnacle coverage on the carapace, plastron, jaw and/or inguinal region was present on 7/35 (20.0%) turtles. Thirty-three physical abnormalities/injuries were noted among 21/35 (60.0%) turtles, with 6/35 (17.1%) turtles having more than one abnormality. Ten (28.6%) presented with capture-induced lesions, predominantly minor abrasions/erythema/bruises to the neck, flippers/claws and/or inguinal region, but which also included four (11.4%) with minor cloacal prolapses that resolved prior to release back into the ocean. Physical trauma not associated with capture was the most prevalent observation noted among 21 turtles (including four with capture-induced observations) and consisted of four (11.4%) with notches in the marginal scutes, right dorsal maxilla or ventral left mandible; four (11.4%) with rake marks or healed/healing injuries likely from interactions with sharks; three (8.6%) with scars above the left eye, around the midline carapace, or the marginal scutes; and two (5.7%) with indentations in the marginal scutes or at the base of the skull. Four (11.4%) turtles also exhibited erosion of the marginal scutes and/or jaw as a result of barnacles, and two (5.7%) turtles exhibited presumed developmental deformities such as a vertebral scute bump and an extra marginal scute. All the described observations in included study animals were considered representative of the population captured by trawling in a group exposed to similar capture and handling conditions, in addition to acceptable variability in free-ranging sea turtles.

### Reference intervals

Hemolysis was absent in all samples, while mild (1+) lipemia was detected in one sample, which is considered insufficient to cause interference with dry chemistry analysis (Andreasen *et al.*, 1997; Stacy and Innis, 2017; Stacy *et al*., 2019). Measures of central tendency, range and RIs in SI units are reported in Table 2. Supplemental Table 1 reports the same values in conventional units. Two turtles were removed from calculation of RIs: (1) the adult female, as this individual was the only mature turtle captured and her blood biochemical analytes skewed much of the health data to the right; (2) the turtle with severe monofilament line ingestion and low BCI showing evidence of hyporexia based on chemistry results.

**Table 2:** Measures of central tendency, range and reference intervals (with 90% confidence intervals for upper and lower limits) for packed cell volume and plasma biochemical data (including protein electrophoresis) in standard international units for in-water, immature Kemp’s ridley sea turtles (*Lepidochelys kempii*) from Georgia, USA. Parametric methods for sample sizes ≥20 but <40 were used to calculate reference intervals (Friedrichs *et al*., 2012), unless otherwise indicated in the footnotes. Normality was assessed using the Shapiro–Wilk test (Shapiro and Wilk, 1965), while outliers were detected using the Dixon–Reed test (Reed *et al*., 1971). All plasma samples were free of hemolysis and lipemia, except for one sample with mild (1+) lipemia, which is not considered to cause interference using dry chemistry analysis (Andreasen *et al*., 1997; Stacy and Innis, 2017; Stacy *et al*., 2019). Abbreviations: CI, confidence interval; LRL, lower reference limit; RI, reference interval; SD, standard deviation; URL, upper reference limit

Analyte	Mean ± SD	Median	Range	*N*	RI	LRL 90% CI	URL 90% CI
*Hematology*							
Packed cell volume [L L^−1^]	0.32 ± 0.05	0.31	0.20–0.44	33	0.23–0.41	0.21–0.25	0.39–0.43
*Biochemistry*							
Alkaline phosphatase [U L^−1^]	119 ± 54	108	40–344	34	52–232^a^	44–63^a^	193–280^a^
Amylase [U L^−1^]	461 ± 86	473	229–622	34	293–629	250–335	587–671
Aspartate aminotransferase [U L^−1^]	185 ± 51	175	130–427^b^	34	122–233^b^	108–136^b^	219–247^b^
Blood urea nitrogen [mmol L^−1^]	25.5 ± 4.8	24.8	17.5–38.6	34	16.1–34.9	13.7–18.5	32.5–37.3
Calcium [mmol L^−1^]	2.4 ± 0.2	2.4	2.0–2.9	34	1.9–2.8	1.8–2.1	2.7–3.0
Calcium:phosphorus ratio	0.88 ± 0.13	0.86	0.65–1.19	34	0.63–1.13	0.57–0.69	1.07–1.19
Chloride [mmol L^−1^]	124 ± 5	124	115–139	34	115–134	112–117	132–136
Cholesterol [mmol L^−1^]	2.7 ± 0.5	2.6	1.6–4.2	34	1.6–3.7	1.4–1.9	3.4–4.0
Creatine phosphokinase [U L^−1^]	1513 ± 865	1239	784–4513	34	510–2955^c^	395–693^c^	2187–3812^c^
Gamma glutamyl transferase [U L^−1^]	–	<5	<5–7	34	–	–	–
Glucose (plasma) [mmol L^−1^]	6.8 ± 1.1	6.7	4.7–9.2	34	4.7–8.9	4.1–5.2	8.4–9.4
Lipase [U L^−1^]	22 ± 18	14	1–65^d^	32	4–71^a,d^	3–6^a.d^	49–104^a,d^
Magnesium [mmol L^−1^]	2.3 ± 0.2	2.3	1.9–2.8	34	1.9–2.8	1.7–2.0	2.7–2.9
Phosphorus [mmol L^−1^]	2.8 ± 0.4	2.7	1.9–3.7	34	2.0–3.5	1.8–2.2	3.3–3.7
Potassium [mmol L^−1^]	4.9 ± 0.3	5.0	4.3–5.5	34	4.3–4.6	4.1–4.5	5.4–5.7
Sodium [mmol L^−1^]	164 ± 4	164	154–175	34	155–172	153–157	170–174
Triglycerides [mmol L^−1^]	1.1 ± 0.5	1.1	0.4–2.7	34	0.4–2.5^a^	0.3–0.5^a^	2.0–3.1^a^
Uric acid [μmol L^−1^]	103.0 ± 31.2	107.6	41.6–178.4	34	41.9–164.2	26.5–57.3	148.8–178.0
*Total solids and protein electrophoresis*							
Total protein [g L^−1^]	38 ± 6	38	24–52	34	26–50	23–29	47–53
Total solids [g L^−1^]	37 ± 6	38	24–50	34	25–49	22–28	46–52
Pre-albumin [g L^−1^]	2.2 ± 1.5	1.7	0.7–6.4	34	0.6–5.7^a^	0.5–0.8^a^	4.3–7.5^a^
Albumin [g L^−1^]	7.8 ± 1.7	7.6	4.8–12.2	34	4.5–11.0	3.7–5.4	10.2–11.8
Alpha_1_-globulins [g L^−1^]	3.3 ± 1.1	3.5	1.3–5.2	34	1.2–5.5	0.6–1.7	4.9–6.0
Alpha_2_-globulins [g L^−1^]	4.3 ± 1.1	4.2	2.4–7.7	34	2.1–6.4	1.6–2.6	5.9–7.0
Beta-globulins [g L^−1^]	9.3 ± 2.6	8.9	5.0–19.4	34	5.5–14.8^a^	4.9–6.2^a^	13.0–16.7^a^
Gamma-globulins [g L^−1^]	10.7 ± 2.8	10.3	5.3–17.5	34	5.4–16.1	4.0–6.7	14.8–17.4
Total globulins [g L^−1^]	27.6 ± 5.0	28.0	17.4–39.8	34	17.9–37.4	15.5–20.4	34.9–40.0
Albumin:globulin ratio	0.37 ± 0.07	0.37	0.21–0.53	34	0.22–0.51	0.19–0.26	0.48–0.55

a ^a^Reference intervals were calculated using logarithmic transformations, as original data were non-normal.

b ^b^427 U L^−1^ was an outlier; this value was removed from reference interval calculations. The second highest value was 246 U L^−1^.

c ^c^Reference intervals were calculated using the robust method with a logarithmic transformation, as data could not be transformed to meet the assumptions of normality for parametric methods.

d ^d^1 U L^−1^ was an outlier; this value was removed from reference interval calculations. The second lowest value was 5 U L^−1^.

**Figure 2:**
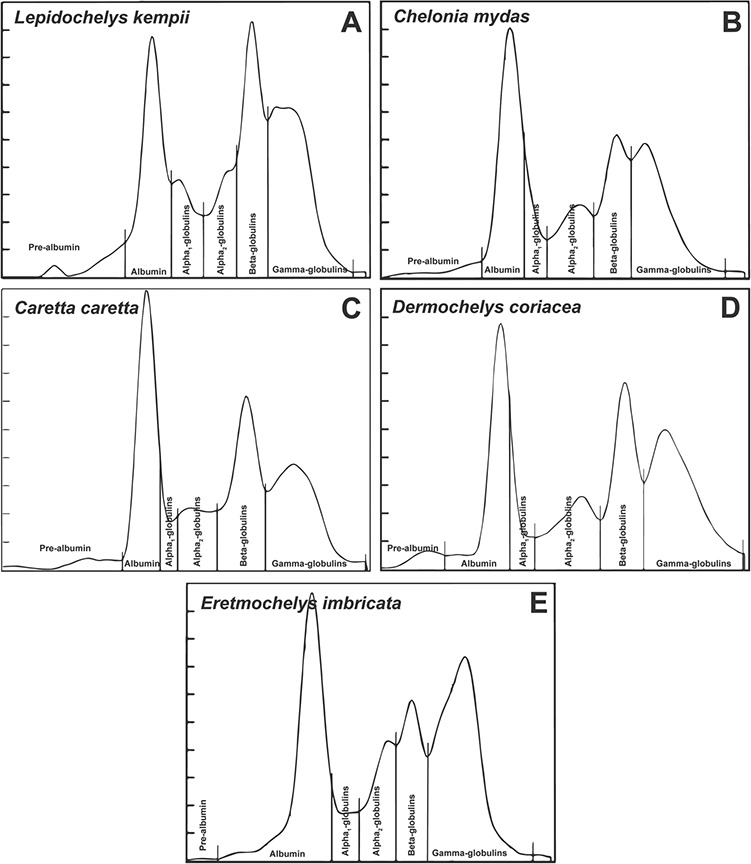
Representative plasma protein electrophoretograms of five immature, free-ranging sea turtle species based on identical laboratory methodology: (a) Kemp’s ridley sea turtle, *Lepidochelys kempii* (SCL_min_: 45.8 cm; this study), (b) green turtle, *Chelonia mydas* (SCL_min_: 25.9 cm; captured in Florida’s Big Bend, USA; Perrault, unpublished data), (c) loggerhead sea turtle, *Caretta caretta* (SCL_min_: 33.1 cm; captured in Florida’s Big Bend, USA; Perrault, unpublished data), (d) leatherback sea turtle, *Dermochelys coriacea* (curved carapace length: 134.3 cm; captured off of Shackleford Banks, NC, USA; Harms, unpublished data) and (e) hawksbill sea turtle, *Eretmochelys imbricata* (SCL_min_: 36.0 cm; captured off of Key West, FL, USA; Wood, unpublished data), showing the fractions of interest: pre-albumin, albumin, alpha_1_-globulins, alpha_2_-globulins, beta globulins and gamma-globulins. By convention, no units are reported on the y-axis (Gicking *et al*., 2004).

A representative electrophoretogram for Kemp’s ridleys is presented in Fig. 2 along with representative electrophoretograms from four other immature, foraging sea turtle species for comparison. All individuals included in Fig. 2 were analyzed using the same laboratory and analytical methods as this study.

 A very strong positive relationship (*r^2^* = 0.86; *P* < 0.001; *N* = 35) existed between total solids and total protein (both in g L^−1^), with the relationship described by the formula:

$$ \mathrm{Total}\ \mathrm{solids}=\left(1.032\times \mathrm{total}\ \mathrm{protein}\right)\hbox{--}\ 0.945 $$

### Correlations with Size

Several blood analytes showed significant positive or negative relationships with SCL_min_ as determined by linear regression. PCV (*r^2^* = 0.69; *P* < 0.001; *N* = 32), amylase (*r^2^* = 0.55; *P* < 0.001; *N* = 34), calcium:phosphorus ratio (*r^2^* = 0.19; *P* = 0.011; *N* = 33), cholesterol (*r^2^* = 0.17; *P* = 0.017; *N* = 33), magnesium (*r^2^* = 0.16; *P* = 0.020; *N* = 33), triglycerides (*r^2^* = 0.14; *P* = 0.030; *N* = 33), total solids (*r^2^* = 0.47; *P* < 0.001; *N* = 34), total protein (*r^2^* = 0.48; *P* < 0.001; *N* = 34), pre-albumin (*r^2^* = 0.13; *P* = 0.039; *N* = 34), albumin (*r^2^* = 0.28; *P* = 0.001; *N* = 34), alpha_1_-globulins (*r^2^* = 0.29; *P* = 0.001; *N* = 34), alpha_2_-globulins (*r^2^* = 0.16; *P* = 0.021; *N* = 34), beta-globulins (*r^2^* = 0.15; *P* = 0.022; *N* = 34), gamma-globulins (*r^2^* = 0.23; *P* = 0.005; *N* = 34) and total globulins (*r^2^* = 0.34; *P* < 0.001; *N* = 34) showed a significant positive relationship with SCL_min_, while AST (*r^2^* = 0.15; *P* = 0.025; *N* = 33) and chloride (*r^2^* = 0.23; *P* = 0.005; *N* = 33) showed a significant negative relationship with SCL_min_ (see Supplemental Table 2 for complete statistical results).

**Figure 3:**
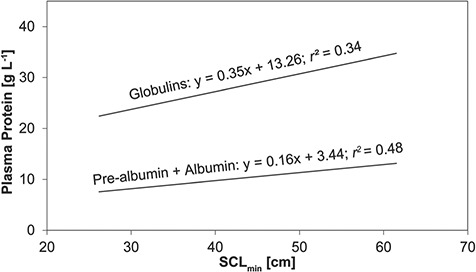
Lines of best fit from the linear regression of pre-albumin + albumin and globulins in comparison to minimum straight carapace length (SCL_min_) in Kemp’s ridley sea turtles (*Lepidochelys kempii*) from Georgia, USA. The slope of pre-albumin + albumin was significantly lower than the slope of globulins.

The slopes of the lines of best fit comparing pre-albumin + albumin (m = 0.16) and total globulins (m = 0.35) in relation to SCL_min_ were significantly different (*t*(64) = −2.11; *P* = 0.008) (Fig. 3).

## Discussion

This study reports blood analyte RIs and length-specific correlations for immature Kemp’s ridley sea turtles from an in-water study in Georgia, USA. In addition to filling a knowledge gap for basic clinicopathological data for this life stage, geographical region and season (spring/summer) of this species, the data herein contribute to understanding blood analyte changes in association with presumptive somatic growth and dietary shifts, which will be useful for interpretations on an individual and population level. Trawl times in this study were kept to a minimum (≤30 mins) to reduce the impacts of forced submergence; however, possible effects on blood analytes associated with the time frame and method of animal capture utilized in this study cannot be ruled out (Stabenau *et al*. 1991; Gregory *et al.*, 1996; Stabenau and Vietti, 2003). We observed minimal evidence of capture stress, as glucose concentrations in only 5/34 turtles (14.7%) were >8.3 mmol/L (>150 mg/dL), a cut-off value considered suggestive of hyperglycemia as compared to previous investigations of clinically convalescent Kemp’s ridleys (Turnbull et al., 2000; Innis et al., 2007, 2009). Additionally, in rehabilitating Kemp’s ridleys and green turtles in North Carolina, USA, significant changes in glucose concentrations were not observed 2 h after a meal (Anderson *et al*., 2011). Despite this finding, feeding immediately before capture may also have contributed to slight plasma glucose variations in Kemp’s ridleys from this study.

### Physical examination and morphometrics

For the establishment of clinically relevant RIs, the population of interest must be apparently healthy (Walton, 2001; Osborne *et al.*, 2010). The animals included in this study were assumed to be in ‘good health’ at time of capture and representative of a free-ranging, in-water caught group based on external physical examination as the turtles (1) had few, mostly minor, external abnormalities, (2) were free of excessive epibiota and (3) all had good BCI. It is possible that some stress effects occurred due to trawling capture methods, as several blood analytes (e.g. CPK, glucose, lactate, LDH, phosphorus) in sea turtles reportedly change significantly in response to increased entanglement times in gillnets (Snoddy *et al.*, 2009); however, trawl times in our study were limited to a maximum of 30 min, which was lower than the study conducted by Snoddy *et al.*, (2009) with soak times ranging from 20–240 min.

The BCI of all turtles in this study ranged from 1.45 to 1.90 (mean ± SE: 1.62 ± 0.02), which overlaps with apparently healthy Kemp’s ridleys of similar size (mean ± SE: 1.56 ± 0.02) captured in Florida’s Big Bend (Perrault *et al.*, 2017). In immature and adult loggerhead turtles that were stranded along the southeastern coast of the United States, BCIs were, on average, 1.09, 1.27 and 1.49 for individuals that stranded dead with chronic debilitation (CD), stranded alive with CD and then began feeding for up to 10 weeks in rehabilitation and for those that recovered from CD (pre-release data), respectively. In that same study, healthy control loggerheads had a mean BCI of 1.56 (Stacy *et al.*, 2018b). Therefore, it can be assumed that the turtles in this study were of good body condition and that our blood analytes and subsequent RIs are likely representative of an apparently healthy population that can serve as a reference for future health-based investigations of Kemp’s ridleys. Subsequent studies should provide descriptions of BCI by species, life-stage class and health status (i.e. emaciated, normal, robust), so that these scores may be used to interpret the results of physical examinations and morphometric measurements (Flint *et al.*, 2010a; Stacy *et al.*, 2018b). For example, one of the excluded turtles of this study with monofilament line ingestion was removed due to a number of measured blood analytes falling on the extreme low or high end of the range in comparison to other study animals.

### Packed cell volume

PCV is an important indicator of health as it provides information on hydration status and anemia (Stamper *et al.*, 2005). The PCV of study turtles ranged from 0.20 to 0.44 L L^−1^ (mean ± SE: 0.32 ± 0.01 L L^−1^), which is similar to mean values reported in other immature Kemp’s ridley studies (Stabenau *et al.*, 1991: 0.31 L L^−1^; Carminati *et al.*, 1994: 0.31 L L^−1^; Innis *et al.*, 2009: 0.30 L L^−1^). We observed a positive relationship between PCV and SCL_min_, a finding that has been documented in six of seven sea turtle species including green, hawksbill, Kemp’s ridley, leatherback, loggerhead and olive ridley sea turtles (*Lepidochelys olivacea*) (Frair, 1977; Wood and Ebanks, 1984; Casal *et al.*, 2009; Rousselet *et al.*, 2013; Perrault *et al.*, 2016b; Stacy *et al.*, 2018a). Red blood cells (RBCs) are known to increase in diameter, number and volume as turtles grow and age (Frair, 1977). It is also presumed that an increased number of circulating RBCs is beneficial for meeting oxygen demands associated with longer dive times in larger turtles, hence the higher PCV in mature life stages (Stamper *et al.*, 2005; Perrault *et al.*, 2016b; Stacy *et al.*, 2018a).

### Electrolytes and minerals

Plasma electrolytes (e.g. sodium, chloride, potassium) and minerals (e.g. calcium, phosphorus, magnesium) of study turtles fell within the normal ranges for other sea turtle species (Stacy and Innis, 2017). Negative size-related changes in electrolytes in sea turtles have been previously observed (Hasbún *et al.*, 1998; Stacy *et al.*, 2018a). The negative association between SCL_min_ and plasma chloride may be associated with changes in diet as these organisms transition from oceanic to neritic habitats. Kemp’s ridleys are described as having an oceanic-neritic developmental pattern (Snover *et al.*, 2007), where they undergo early development in the pelagic environment followed by recruitment back to neritic zones for foraging and reproduction (Collard and Ogren, 1990; Bolten, 2003). This habitat shift likely leads to a dietary shift, whereby oceanic Kemp’s ridleys forage on epipelagic organisms (e.g. gastropods, malacostracans, algae) and then change to a diet of benthic crabs and mollusks in the neritic environment (Shaver, 1991; Jones and Seminoff, 2013). Other potential explanations for this association include osmoregulatory differences that may occur as these animals transition in diets and change their diving behavior or as they experience somatic growth, including that of the salt gland (Stacy *et al.*, 2018a). The observed positive correlations of SCL_min_ with calcium:phosphorus ratio and plasma magnesium are presumptively associated with dietary shifts in addition to phases of somatic growth of this life stage in this region, as lower concentrations of minerals reportedly are typical for immature life-stage classes in comparison to adults (Delgado *et al.*, 2011).

We found no significant trends with turtle size and plasma potassium concentrations. However, potassium should be interpreted in context of possible spurious increases, especially if hemolysis is observed in plasma (Stacy *et al.*, 2019) or based on methodology of turtle capture since trawling can result in rapidly increased potassium due to metabolic acidosis in response to short-term forced submergence (Stabenau *et al.*, 1991). Mean potassium concentrations of Kemp’s ridleys in this study (mean ± SD = 4.9 ± 0.3 mmol L^−1^; range 4.3–5.5 mmol L^−1^) were within previously reported normal limits for this species (Innis *et al.*, 2009; Coleman *et al.*, 2016; Stacy and Innis, 2017; McNally *et al.*, 2020); however, 17/34 (50%) of turtles had values >5.0 mmol L^−1^, with two turtles having potassium concentrations of 5.5 mmol L^−1^. In cold-stunned Kemp’s ridleys, potassium concentrations >5.5 mmol L^−1^ were associated with mortality (Innis *et al.*, 2009; Keller *et al.*, 2012), with concentrations ranging from 5.0 to 5.4 mmol L^−1^ also considered to be abnormally high (Stacy *et al.*, 2013). For healthy, foraging Kemp’s ridleys captured by trawl, underlying mechanisms for hyperkalemia include metabolic acidosis and/or muscle damage from exertion during trawling, and thus slightly elevated potassium concentrations may be less concerning here than with cold-stunned individuals; yet, hyperkalemia should be considered due to potential impacts on cardiac function (Innis *et al.*, 2014).

### Tissue enzyme activities

Generally, plasma enzyme activities (e.g. ALP, AST, GGT) are highly variable in sea turtles and not as tissue specific as in mammals, making interpretation difficult and thereby limiting their diagnostic value (Anderson *et al.*, 2013; Petrosky *et al.*, 2015). Conversely, amylase and lipase are two digestive enzymes with reported high activities in pancreatic tissue; however, the clinical significance of these enzymes in sea turtles remains unknown (Anderson *et al.*, 2013; Petrosky *et al.*, 2015). A moderate positive correlation between SCL_min_ and plasma amylase was observed in Kemp’s ridleys from this study, similar to captive, immature loggerheads from Japan (Kakizoe *et al.*, 2007). Juvenile green turtles reportedly showed significantly higher plasma activities of amylase post-prandially in comparison to baseline values established pre-prandially (Anderson *et al.*, 2011), suggesting that food intake increases amylase activity. The positive size-related trend observed between SCL_min_ and amylase in this study could be attributed to post-prandial increases as the most recent food consumption in study turtles is unknown. Additionally, general dietary and/or water salinity shifts that occur as younger Kemp’s ridleys transition from their oceanic phase to their neritic phase could be at play, as these animals are known to forage on dissimilar food items during these different stages of development (Shaver, 1991; Jones and Seminoff, 2013).

We also observed a weak negative correlation between SCL_min_ and plasma AST activity, a trend that has been documented in immature and mature green turtles from Taiwan (Fong *et al.*, 2010). This enzyme has low organ specificity and showed activities in 19/30 (63.3%) and 9/9 (100%) different tissues of loggerheads and Kemp’s ridleys, respectively, with highest tissue activities observed in liver, kidney, pancreas and cardiac/skeletal muscle (Anderson *et al.*, 2013; Petrosky *et al.*, 2015). Normal plasma AST activity for reptiles is <250 U L^−1^ and all Kemp’s ridleys in this study, except one (427 U L^−1^, an outlier), had values below this threshold (Campbell, 2006). Increased AST activities in clinical settings can suggest hepatic or muscular injury, but due to its low organ specificity, other conditions must be considered (Campbell, 2006; Anderson *et al.*, 2013). A possible explanation for the negative correlation between AST activity and SCL_min_ includes faster tissue growth at smaller body sizes (Stacy and Innis, 2017; Stacy *et al.*, 2018a), with a gradual slowing of growth rate as turtles continue to mature (Chaloupka and Zug, 1997; Bolten, 2003).

### Lipids

The observed positive size-related correlations in Kemp’s ridleys between cholesterol and triglycerides and SCL_min_ were expected (Day and Alexander, 1974; Morpurgo and Gelman, 1991; Hasbún *et al.*, 1998; Labrada-Martagón *et al.*, 2010; Delgado *et al.*, 2011; Rousselet *et al.*, 2013). Similar to other analytes, these size-related changes are presumably associated with dietary shifts (Shaver, 1991; Jones and Seminoff, 2013; Stacy *et al.*, 2018a). Changes in plasma lipids as a result of nutritional alterations occurring during seasonal changes have also been documented in other reptiles (Morpurgo and Gelman, 1991; Labrada-Martagón *et al.*, 2010).

### Protein electrophoresis and total solids

Plasma protein fractionation has been evaluated in just two studies of ridley turtles to date, both in Kemp’s ridleys from the eastern Gulf of Mexico in Florida’s Big Bend; however, RIs were not established due to low sample sizes and the presence of a harmful algal bloom (HAB) at the time of sampling (Perrault *et al.*, 2014, 2017). Perrault *et al.* (2017) used the same laboratory and analytical methodology as this study to analyze for plasma proteins in Kemp’s ridleys from the Big Bend. Results for total protein and all protein fractions were similar between the two populations with the exception of the gamma-globulins, which were 5 g/L lower in turtles from this study. This can be explained by the red tide (HAB) bloom and its associated brevetoxins that were present near the time of sampling in the Big Bend, as a number of protein fractions, including gamma-globulins, have been shown to be positively correlated with brevetoxin exposure in manatees (*Trichechus manatus*), loggerheads and green turtles (Walsh *et al.*, 2015, 2019; Perrault *et al.*, 2016a, 2017). Other potential explanations for this dissimilarity include additional differences in antigenic stimulation (e.g. parasite burden) between the two populations (Osborne *et al.*, 2010).

As expected, all plasma proteins showed significant positive correlations with SCL_min_. Other sea turtle studies have noted similar size-related increases and suggest that these changes are related to dietary shifts, immune stimulation, physiological changes associated with vitellogenesis and/or somatic growth (Frair and Shah, 1982; Kakizoe *et al.*, 2007; Casal *et al.*, 2009; Osborne *et al.*, 2010; Rousselet *et al.*, 2013; Espinoza-Romo *et al.*, 2018; Stacy *et al.*, 2018a).

Globulins (alpha-, beta- and gamma-globulins) show variations more frequently than albumin (often with pre-albumin included) in sea turtles (Stacy *et al.*, 2018a). Size-related increases in plasma globulin concentrations are presumably related to nutritional alterations and/or increased exposure to antigens as turtles grow and age, especially as they recruit back to neritic habitats where more frequent antigen exposure occurs (Innis *et al.*, 2010; Stacy *et al.*, 2018a). This is further confirmed by the increasing trend of beta- and gamma-globulins with SCL_min_ as both of these protein fractions are known to contain immunoglobulins and various acute phase proteins (Osborne *et al.*, 2010; Evans, 2011). Globulins in Kemp’s ridleys from this study showed a rate of increase 2.2 times that of pre-albumin + albumin. Plasma globulins in juvenile Azorean loggerheads showed a rate of increase 3.6 times that of albumin, further indicating that globulins increase faster than albumin as juvenile turtles undergo somatic growth (Stacy *et al.*, 2018a); however, pre-albumin was not included in those calculations as protein electrophoresis was not conducted in that study. Interestingly, the albumin:globulin ratio did not decrease with increasing SCL_min_ in turtles from this study, as it did in Azorean loggerheads (Stacy *et al.*, 2018a). This is possibly due to the slower rate of increase of globulins versus pre-albumin + albumin seen here in comparison to Azorean loggerheads, although differences in analytical methodology must be considered (Stacy *et al.*, 2018a).

The comparison of immature Kemp’s ridleys electrophoretograms to those from representatives of four other immature sea turtle species in Fig. 2 provides a unique opportunity for visual comparison and shows some similarities, including the presence of a pre-albumin fraction in all species. Some minor variations are observed in alpha-globulins, with more obvious differences in beta- and gamma-globulins. Considerations for these species-specific differences include variations in diet, fibrinogen concentration and antigenic exposure in various habitats.

The gold standard for determination of total plasma protein in sea turtles is the Biuret method, while refractometry provides the ability to rapidly and easily estimate total solids, which correlate with total plasma protein in normal colored samples (Macrelli *et al.*, 2013, Stacy and Innis, 2017). Potential overestimation errors in total solid estimates can arise due to confounding factors including hemolysis, lipemia or elevated concentrations of various biochemical analytes (e.g. glucose, urea, sodium, chloride) (Stacy and Innis, 2017). Previous studies of loggerhead and green turtles have documented strong to very strong correlations between total solids and total protein (Bolten and Bjorndal, 1992; Rousselet *et al.*, 2013), similar to our findings. Additionally, our results showed overlapping measures of central tendencies and reference ranges for total solids and total protein (Table 2), similar to leatherback and loggerhead turtles (Deem *et al.*, 2006, 2009). Mammalian conversion equations from total solids to total protein exist; yet, these have not been proven useful in reptiles to date, and species-specific total solid to total protein conversion equations should be developed for sea turtles (Bolten and Bjorndal, 1992).

## Conclusions

Here, we provide morphometric and blood analyte data from immature, actively foraging Kemp’s ridleys from the northwest Atlantic Ocean that were considered representative of the population and that were captured by trawling. Conservation physiology projects like these seek to explore wildlife populations and provide a baseline for future studies investigating their responses to physiological changes, environmental disturbances or disease, so that we may better understand reasons for population declines or changes in population dynamics (Wikelski and Cooke, 2006, Cooke and O’Connor, 2010). The RIs established here, in due consideration of capture technique in this study, provide valuable information for individuals that strand and enter rehabilitation facilities, in addition to future health assessment studies that may be conducted for population monitoring or in response to environmental changes or disturbances (Deem *et al*., 2001; Aguirre and Lutz, 2004; Osborne *et al.*, 2010; Flint *et al*., 2015; Kelly *et al*., 2015; Page-Karjian *et al*., 2015, 2020; Villa *et al.*, 2017; Stacy *et al.*, 2017, 2018a). We identified a number of physiological changes in these organisms considered in ‘good health’ that are likely associated with dietary/habitat shifts, somatic growth and/or other physiological alterations. The information herein provides a better understanding of physiological changes associated with growth in immature Kemp’s ridley sea turtles, which can provide clinical utility for individuals during rehabilitation and in interpretations of blood data in population-level health assessments to help guide conservation and management decisions.

## Supplementary Material

Perrault_et_al_Lk_Health_Assessment_Supplemental_Table_1_coaa091

Perrault_et_al_Lk_Health_Assessment_Supplemental_Table_2_coaa091
