# Supplementary material for: Blood analytes of immature Kemp’s ridley sea turtles (Lepidochelys kempii) from Georgia, USA: reference intervals and body size correlations
Source: Conserv Physiol. 2020 Dec 1;8(1):coaa091. doi: 10.1093/conphys/coaa091 (PMC7720087; doi:10.1093/conphys/coaa091)
Supplement: Perrault_et_al_Lk_Health_Assessment_Supplemental_Table_1_coaa091 [file perrault_et_al_lk_health_assessment_supplemental_table_1_coaa091.docx]

| **Supplemental Table 1**. Measures of central tendency, range, and reference intervals (with 90% confidence intervals for upper and lower limits) for packed cell volume and plasma biochemical data (including protein electrophoresis) in conventional units for in-water, immature Kemp’s ridley sea turtles (*Lepidochelys kempii*) from Georgia USA. Parametric methods for sample sizes ≥20 but <40 were used to calculate reference intervals (Friedrichs *et al*., 2012), unless otherwise indicated in the footnotes. Normality was assessed using the Shapiro-Wilk test (Shapiro and Wilk, 1965), while outliers were detected using the Dixon-Reed test (Reed *et al*., 1971). All plasma samples were free of hemolysis and lipemia, except for one sample with mild (1+) lipemia which is not considered to cause interference using dry chemistry analysis (Andreasen *et al*., 1997; Stacy and Innis, 2017; Stacy *et al*., 2019). Abbreviations: CI, confidence interval; LRL, lower reference limit; RI, reference interval; SD, standard deviation; URL, upper reference limit. | | | | | | | |
| --- | --- | --- | --- | --- | --- | --- | --- |
| **Analyte** | **Mean±SD** | **Median** | **Range** | ***N*** | **RI** | **LRL 90% CI** | **URL 90% CI** |
| *Hematology* |  |  |  |  |  |  |  |
| Packed cell volume [%] | 32±5 | 31 | 20–44 | 33 | 23–41 | 21–25 | 39–43 |
| *Biochemistry* |  |  |  |  |  |  |  |
| Alkaline phosphatase [U L^-1^] | 119±54 | 108 | 40–344 | 34 | 52–232^a^ | 44–63^a^ | 193–280^a^ |
| Amylase [U L^-1^] | 461±86 | 473 | 229–622 | 34 | 293–629 | 250–335 | 587–671 |
| Aspartate aminotransferase [U L^-1^] | 185±51 | 175 | 130–427^b^ | 34 | 122–233^b^ | 108–136^b^ | 219–247^b^ |
| Blood urea nitrogen [mg dL^-1^] | 71±13 | 70 | 49–108 | 34 | 45–98 | 38–52 | 91–104 |
| Calcium [mg dL^-1^] | 9.5±0.9 | 9.5 | 8.1–11.4 | 34 | 7.7–11.3 | 7.3–8.2 | 10.9–11.8 |
| Calcium:phosphorus ratio | 1.14±0.16 | 1.11 | 0.84–1.54 | 34 | 0.82–1.46 | 0.74–0.90 | 1.38–1.54 |
| Chloride [mmol L^-1^] | 124±5 | 124 | 115–139 | 34 | 115–134 | 112–117 | 132–136 |
| Cholesterol [mg dL^-1^] | 103±21 | 101 | 63–161 | 34 | 62–143 | 52–72 | 133–153 |
| Creatine phosphokinase [U L^-1^] | 1513±865 | 1239 | 784–4513 | 34 | 510–2955^c^ | 395–693^c^ | 2187–3812^c^ |
| Gamma glutamyl transferase [U L^-1^] | – | <5 | <5–7 | 34 | – | – | – |
| Glucose (plasma) [mg dL^-1^] | 122±19 | 121 | 85–165 | 34 | 84–160 | 75–94 | 151–170 |
| Glucose (whole blood) [mg dL^-1^] | 149±39 | 144 | 88–244 | 33 | 72–226 | 52–91 | 206–245 |
| Lipase [U L^-1^] | 22±18 | 14 | 1–65^d^ | 32 | 4–71^a,d^ | 3–6^a.d^ | 49–104^a,d^ |
| Magnesium [mg dL^-1^] | 5.6±0.6 | 5.6 | 4.7–6.8 | 34 | 4.5–6.8 | 4.2–4.8 | 6.5–7.1 |
| Phosphorus [mg dL^-1^] | 8.5±1.3 | 8.5 | 5.9–11.5 | 34 | 6.1–10.9 | 5.5–6.7 | 10.3–11.6 |
| Potassium [mmol L^-1^] | 4.9±0.3 | 5.0 | 4.3–5.5 | 34 | 4.3–5.6 | 4.1–4.5 | 5.4–5.7 |
| Sodium [mmol L^-1^] | 164±4 | 164 | 154–175 | 34 | 155–172 | 153–157 | 170–174 |
| Triglycerides [mg dL^-1^] | 100±46 | 98 | 35–238 | 34 | 36–222^a^ | 29–46^a^ | 177–279^a^ |
| Uric acid [mg dL^-1^] | 1.7±0.5 | 1.8 | 0.7–3.0 | 34 | 0.7–2.8 | 0.5–1.0 | 2.5–3.0 |
| *Total solids and protein electrophoresis* | | | | | | | |
| Total protein [g dL^-1^] | 3.8±0.6 | 3.8 | 2.4–5.2 | 34 | 2.6–5.0 | 2.3–2.9 | 4.7–5.3 |
| Total solids [g dL^-1^] | 3.7±0.6 | 3.8 | 2.4–5.0 | 34 | 2.5–4.9 | 2.2–2.8 | 4.6–5.2 |
| Pre-albumin [g dL^-1^] | 0.22±0.15 | 0.17 | 0.07–0.64 | 34 | 0.06–0.57^a^ | 0.05–0.08^a^ | 0.43–0.75^a^ |
| Albumin [g dL^-1^] | 0.78±0.17 | 0.76 | 0.48–1.22 | 34 | 0.45–1.10 | 0.37–0.54 | 1.02–1.18 |
| Alpha_1_-globulins [g dL^-1^] | 0.33±0.11 | 0.35 | 0.13–0.52 | 34 | 0.12–0.55 | 0.06–0.17 | 0.49–0.60 |
| Alpha_2_-globulins [g dL^-1^] | 0.43±0.11 | 0.42 | 0.24–0.77 | 34 | 0.21–0.64 | 0.16–0.26 | 0.59–0.70 |
| Beta-globulins [g dL^-1^] | 0.93±0.26 | 0.89 | 0.50–1.94 | 34 | 0.55–1.48^a^ | 0.49–0.62^a^ | 1.30–1.67^a^ |
| Gamma-globulins [g dL^-1^] | 1.07±0.28 | 1.03 | 0.53–1.75 | 34 | 0.54–1.61 | 0.40–0.67 | 1.48–1.74 |
| Total globulins [g dL^-1^] | 2.76±0.50 | 2.80 | 1.74–3.98 | 34 | 1.79–3.74 | 1.55–2.04 | 3.49–4.00 |
| Albumin:globulin ratio | 0.37±0.07 | 0.37 | 0.21–0.53 | 34 | 0.22–0.51 | 0.19–0.26 | 0.48–0.55 |
| ^a^ Reference intervals were calculated using logarithmic transformations, as original data were non-normal.  ^b^ 427 U L^-1^ was an outlier; this value was removed from reference interval calculations. The second highest value was 246 U L^-1^.  ^c^ Reference intervals were calculated using the robust method with a logarithmic transformation, as data could not be transformed to meet the assumptions of normality for parametric methods.  ^d^ 1 U L^-1^ was an outlier; this value was removed from reference interval calculations. The second lowest value was 5 U L^-1^. | | | | | | | |
