# Supplementary material for: Blood analytes of immature Kemp’s ridley sea turtles (Lepidochelys kempii) from Georgia, USA: reference intervals and body size correlations
Source: Conserv Physiol. 2020 Dec 1;8(1):coaa091. doi: 10.1093/conphys/coaa091 (PMC7720087; doi:10.1093/conphys/coaa091)
Supplement: Perrault_et_al_Lk_Health_Assessment_Supplemental_Table_2_coaa091 [file perrault_et_al_lk_health_assessment_supplemental_table_2_coaa091.docx]

| **Supplemental Table 2**. Significant linear regressions between minimum straight carapace length (SCL_min_) and the measured blood analytes in Kemp’s ridley sea turtles (*Lepidochelys kempii*) from Georgia USA. | | | | | | |
| --- | --- | --- | --- | --- | --- | --- |
| Analyte | Regression equation | Slope 95% CI | Intercept 95% CI | *r^2^* | *P* | *N* |
| SCL_min_ v packed cell volume | y = 0.43x + 14.36 | 0.32, 0.54 | 9.68, 19.05 | 0.69 | <0.001 | 32 |
| SCL_min_ v amylase^a^ | y = 7.23x + 159.74 | 4.89, 9.56 | 59.23, 260.25 | 0.55 | <0.001 | 34 |
| SCL_min_ v aspartate aminotransferase^a^ | y = -1.23x + 228.87 | -2.29, -0.16 | 183.48, 274.26 | 0.15 | 0.025 | 33 |
| SCL_min_ v calcium:phosphorus | y = 0.01x + 0.60 | 0.002, 0.01 | 0.39, 0.82 | 0.19 | 0.011 | 33 |
| SCL_min_ v chloride^a^ | y = -0.21x + 133.02 | -0.36, -0.07 | 126.87, 139.16 | 0.23 | 0.005 | 33 |
| SCL_min_ v cholesterol^a^ | y = 0.03x + 1.55 | 0.01, 0.05 | 0.64, 2.45 | 0.17 | 0.017 | 33 |
| SCL_min_ v magnesium | y = 0.01x + 1.86 | 0.002, 0.02 | 1.47, 2.26 | 0.16 | 0.020 | 33 |
| SCL_min_ v triglycerides^a^ | y = 0.02x + 0.14 | 0.002, 0.05 | -0.77, 1.04 | 0.14 | 0.030 | 33 |
| SCL_min_ v total solids | y = 0.55x + 14.40 | 0.34, 0.76 | 5.37, 23.42 | 0.47 | <0.001 | 34 |
| SCL_min_ v total protein | y = 0.51x + 16.59 | 0.32, 0.70 | 8.38, 24.80 | 0.48 | <0.001 | 34 |
| SCL_min_ v pre-albumin^b^ | y = 0.01x - 0.13 | 0.001, 0.02 | -0.52, 0.27 | 0.13 | 0.039 | 34 |
| SCL_min_ v albumin | y = 0.10x + 3.55 | 0.04, 0.16 | 1.02, 6.08 | 0.28 | 0.001 | 34 |
| SCL_min_ v alpha_1_-globulins | y = 0.09x – 0.26 | 0.04, 0.14 | -2.43, 1.92 | 0.29 | 0.001 | 34 |
| SCL_min_ v alpha_2_-globulins^b^ | y = 0.01x + 0.41 | 0.001, 0.01 | 0.22, 0.59 | 0.16 | 0.021 | 34 |
| SCL_min_ v beta-globulins^b^ | y = 0.01x + 0.76 | 0.001, 0.01 | 0.58, 0.93 | 0.15 | 0.022 | 34 |
| SCL_min_ v gamma-globulins^a^ | y = 0.13x + 4.85 | 0.04, 0.23 | 0.90, 8.79 | 0.23 | 0.005 | 33 |
| SCL_min_ v total globulins | y = 0.35x + 13.26 | 0.17, 0.53 | 5.70, 20.83 | 0.34 | <0.001 | 34 |
| ^a^ One outlier was removed prior to regression analyses.  ^b^ Log-transformations of pre-albumin, alpha_2_-globulins, and beta-globulins were carried out prior to regression analyses to normalize residuals. | | | | | | |
